# A Survey of Skin Disease among Patients in an Australian Nursing Home

**DOI:** 10.2188/jea.12.336

**Published:** 2007-11-30

**Authors:** Derek Richard Smith, Ron Atkinson, Sa Tang, Zentaro Yamagata

**Affiliations:** 1 Department of Health Sciences, Yamanashi Medical University.; 2 Faculty of Sciences, The University of Southern Queensland.

**Keywords:** Australia, nursing home, prevalence, skin disease, risk factors

## Abstract

Although the number of nursing homes is increasing in Australia, few studies have investigated the dermatologic condition of their patients. To address this issue, we conducted one of the first skin disease investigations of nursing home residents in Queensland, Australia. Methods: Our predominant data source was the attending physicians’ medical reports, which are updated monthly following their physical examination of each patient. Specialist podiatrists’ monthly progress notes were also used as were daily nursing reports. Results: Just over half the patients (54.4%) had at least one skin disease registered among their medical records. Xerosis (dry skin) was the most common affliction, affecting 29.5% of the patients, followed by onychomycosis (tinea unguium) at 22.5% and dermatitis (8.9%). Skin cancer was recorded in 4.9%, while excoriation (3.1%) and unspecified keratosis (2.2%) were slightly less common. Being bedridden was identified as a risk factor for both xerosis (OR 3.9, 95% CI 1.8-8.7) and onychomycosis (OR 18.0, 95% CI 7.5-49.0). Conclusion: Overall, our research suggests that skin diseases are reasonably common among Australian nursing home patients. The presence of certain dermatologic conditions differed from other reports.

Continuing advances in public health and medical care have prolonged lifespan and expanded the elderly demographic worldwide. Older Australians are also on the increase, with people over the age of 65 years currently comprising 12.2% of the national population.^1^ Among them, 8.5% require some degree of nursing care, representing a group of about 190,000 people. To care for them, there are around 140,000 nursing home beds in Australia of various descriptions.^2^ Although skin disease is almost ubiquitous within elderly populations,^3^ their dermatological needs are often unmet and epidemiologic investigations are rare. To address these two public health shortfalls, we conducted one of the first dermatologic audits of nursing home patients within the fast growing region of south east Queensland in Australia.

## METHODS

This study involved a retrospective epidemiologic analysis of skin disease among patients of a large nursing home complex in Queensland, Australia. Our research was initially reviewed and approved by the ethical committee of the University of Southern Queensland in November 2000. All fieldwork was undertaken in November and December 2000, during the tropical summer season in south east Queensland. All medical records were selected and then individually examined to ascertain the presence of skin disease within the past 3 months. There were 4 main data sources from which we obtained the information. The first involved physicians’ medical notes for each patient, which are updated following physical examinations approximately once every month. The second involved podiatrists’ reports which are also updated after their monthly visit and physical examination. Nurses’ progress notes are written daily for each patient and were used, as were interviews with health care staff regarding the skin integrity of individual patients. All data was entered into a spreadsheet program before being analyzed by statistical software. Descriptive statistics for the prevalence of skin disease were calculated, with gender differences in skin disease prevalence investigated using a chi square tests. The relationship between systemic diabetes mellitus and the presence of onychomycosis was also investigated using the chi square test. Multiple logistic regression analyses using patient variables was conducted to derive potential risk factors for dermatological conditions. Odds ratios were adjusted for potential confounding variables such as age, sex, duration of stay in the nursing home and the presence of systemic diabetes mellitus. Calculated p values under 0.05 were regarded as statistically significant throughout.

## RESULTS

For this study we recruited 381 available residents in the nursing home and successfully examined the medical records of 360 (94.5%). Patients were divided between a high-care nursing home environment and medium-care hostel accommodation at a care ratio of 1 to 2.1. As shown in Table 1, most were female (75.8%), with an average age of 84.1 years (SD=9.0) and an age range of 34 to 103 years. Females outnumbered males in every age category and were generally older (mean age 84.8 versus 81.7 years). There were very few alcohol drinkers (4.7%) and tobacco smokers (2.2%) among the group. Their duration of stay in this facility varied from 0.25 to 232 months, with an average stay of 34.9 months (SD=38.1), or approximately 2.9 years each. The median duration of stay was 20 months. Exactly half of the group (50.0%) were aged between 80 and 90 years. Very old patients (over 90 years of age) constituted just over one-quarter of the population (27.7%). Most could communicate effectively (76.9%), although ambulation varied from fully mobile (44.7%) to reliance on a walking aid of some description (46.7%) such as a wheelchair or ‘wheelie-walker’. A small percentage (8.6%) were bedridden. Almost all the patients (96.1%) had at least one underlying disease, while 79.4% suffered multiple systemic diseases. The common systemic diseases recorded were dementia (36.1%), heart disease (25.6%), hypertension (21.4%), stroke (18.6%), arthritis (16.1%), and diabetes mellitus (15.0%). Underlying disease prevalence was virtually identical between the genders.

**Table 1.** Patient demographics.

	n	(%) ^a^
Major characteristics		
Female gender	273	(75.8)
Full communication	277	(76.9)
Smokes tobacco	8	(2.2)

Ambulation ability		
Fully mobile	161	(44.7)
Assistance required	168	(46.7)
Bedridden	31	(8.6)

Mean ± SD		
Age (years)	84.1 ± 9.0	
Duration (months) ^b^	34.9 ± 38.1	

Median		
Duration (months) ^b^	20.0	

^a^ percentage of all patients (n=360)

^b^ duration of stay in the nursing home

Just over half of the patients (54.4%) had at least one skin disease noted in their medical records, while 28.1% had 2 or more skin diseases. The average number of skin diseases among this group was 1.6 (SD=0.8). Xerosis was the most common affliction, affecting 29.5% of the patients, followed by onychomycosis (22.5%) and dermatitis (8.9%) (Table 2). Skin cancer, including basal cell carcinoma, squamous cell carcinoma and melanoma, was recorded among 4.9% of patients, followed by excoriation (3.1%) and unspecified keratosis (2.2%). All other detected skin disease categories affected less than 2% of the total.

**Table 2.** Skin disease prevalence by sex.

	All		Male		Female		p value ^d^

	n	(%) ^a^	n	(%) ^b^	n	(%) ^c^	
Xerosis	106	(29.5)	29	(33.3)	77	(28.2)	0.36
Onychomycosis	81	(22.5)	18	(20.7)	63	(23.1)	0.64
Dermatitis	32	(8.9)	6	(6.9)	26	(9.5)	0.45
Skin cancer	14	(4.9)	5	(5.7)	9	(3.3)	0.30
Excoriation	11	(3.1)	1	(1.1)	10	(3.7)	0.24
Keratosis	8	(2.2)	1	(1.1)	7	(2.6)	0.44
Tinea pedis	6	(1.7)	1	(1.1)	5	(1.8)	0.67
Purpura	6	(1.7)	1	(1.1)	3	(1.1)	0.97

^a^ percentage of all patients (n=360)

^b^ percentage of all males (n=87)

^c^ percentage of all females (n=273)

^d^ gender differences in skin disease prevalence investigated using chi square tests.

The gender distribution of each skin disease was variable when sex-specific prevalence was compared. Males had a higher prevalence of xerosis and skin cancer than females (33.3% versus 28.2% and 5.7% versus 3.3% respectively). Conversely, females suffered higher rates of onychomycosis and dermatitis than males (23.1% versus 20.7 and 9.5% versus 6.9% respectively). None of these gender differences in skin disease prevalence rates were statistically significant however. The most common site for skin disease was the lower leg, with almost three-quarters of all xerosis cases (72.6%) diagnosed in this location (Table 3). The lower arm was another common xerosis site (45.3% of all xerosis cases). All incidences of onychomycosis were detected on the feet (100.0%), while more than one-third of the dermatitis sufferers (37.5%) had dorsal thoracic lesions. A large proportion of the skin cancers diagnosed (42.5%) were located on the patients’ face.

**Table 3.** Skin disease prevalence by location a

	Xerosis		Onychomycosis		Dermatitis		Skin cancer	

	n	(%)	n	(%)	n	(%)	n	(%)
Upper body								
Neck	17	(16)	0		1	(3)	1	(7)
Face	37	(35)	0		5	(16)	6	(43)
Scalp	19	(18)	0		1	(3)	3	(21)

Trunk								
Chest	16	(15)	0		3	(9)	1	(7)
Back	24	(23)	0		12	(38)	1	(7)
Groin	14	(13)	0		3	(9)	0	

Arms								
Upper arms	24	(23)	0		3	(9)	2	(14)
Lower arms	48	(45)	0		5	(16)	2	(14)
Hands	17	(16)	0		0		3	(21)

Legs								
Upper legs	29	(27)	0		1	(3)	2	(14)
Lower legs	77	(73)*	0		2	(6)	2	(14)
Feet	9	(9)	81	(100)	0		1	(7)

^a^ percentage of cases for each category (for example *73% of all xerosis cases were found on the lower leg of patients). The total for each category being 106, 81, 32, and 14 respectively.

Only one skin disease risk factor was identified by multiple logistic regression models in this study. As shown in Table 4, being bedridden was statistically associated with the presence of xerosis and onychomycosis; even when adjusted for confounding variables such as age, sex, duration of stay within the nursing home and the presence of diabetes mellitus. Bedridden status increased the risk of xerosis 3.9 fold (95% CI; 1.8 - 8.7) among our patients. The risk of onychomycosis was also increased, with bedridden patients being 18.0 times more likely to have onychomycosis than mobile patients within this nursing home (95% CI; 7.5 - 49.0). On its own, the presence of systemic diabetes mellitus was not statistically related to the presence of onychomycosis (p=0.684). Similarly, risk factors for other skin diseases were inconclusive and statistically insignificant.

**Table 4.** Risk factors associated with specific skin diseases

Skin disease	Risk Factor	Category	(%) ^a^	p	Odds ratio (95% CI) ^b^
Xerosis	Bedridden	No	(95.0)	-	1.0
		Yes	(5.0)	0.0005	3.94 (1.83 - 8.74)
Onychomycosis	Bedridden	No	(93.3)	-	1.0
		Yes	(6.7)	<0.0001	18.04 (7.55 - 49.04)

^a^ Percentage of all patients (n=360) in that particular subcategory (ie. suffering from xerosis and being bedridden).

^b^ Odds ratios derived from multiple logistic regression using the presence of xerosis and onychomycosis as the dependent variable and patient risk factors as the independent variables (adjusted for age, sex, duration of stay in the nursing home and the presence of diabetes mellitus).

## DISCUSSION

Intrinsic changes within the skin predispose elderly people to a wide variety of cutaneous diseases. Decreasing subcutaneous and dermal thickness compromises the skin’s protective ability, while at a systemic level antigen levels and inflammatory responses are also diminished. These intrinsic reductions compromise the skin’s natural defences, while extrinsic factors such as cumulative sun exposure further degrade the durability of the skin.^4^

Senile xerosis usually results from an age related decrease of stratum comeum lipids over time.^5^ In younger skin, these compounds bind to water molecules and hold them within the sub-dermal matrix, helping to maintain surface moisture and skin integrity.^6^ The natural moisturising factor is another important water-holding compound that also decreases with age. Deficiencies of its metabolic precursor Filaggrin, have been shown in the lower leg of elderly citizens.^7^ Bedridden status is known to significantly reduce the stratum comeum water content among older people.^8^ Furthermore, long-term vitamin C insufficiency may also lead to dermatological abnormalities such as dry, scaly skin.^9^ Our study revealed xerosis as the most common skin disease present among the patients studied, affecting almost one-third of them (29.5%). This result is much lower than other studies of elderly populations, where xerosis prevalences of 38.9% to 85.1% have been recorded.^3^^,^^10^ We suspect these differences relate to different methods of data collection. In previous studies the authors specifically examined the patients’ skin for the presence of dermatological abnormality, while their cohort was possibly biased in a positive direction as it consisted of elderly volutneers^3^ or dermatology clinic outpatients.^10^ In our study, we consulted medical records written by physicians who may not have noted all the patient’s skin problems during their consultation. Furthermore, our cohort comprised all members of the nursing home, regardless of their skin condition. Statistical analysis of the data revealed bedridden status as a significant risk factor for xerosis (OR 3.9), which probably relates to the cascade of systemic ailments leading to bedridden status, of which xerosis is simply a by-product.

Onychomycosis (tinea unguium) is a common superficial disease of human beings and one that often shows increasing prevalence with advanced age.^11^^-^^13^ Diabetes prevalence also increases with age and is known to be a risk factor for fungal disease complications.^14^ Our research revealed onychomycosis affecting almost one-quarter (22.5%) of all patients. A previous study of elderly people has documented a much lower onychomycosis prevalence rate of 10.1%.^15^ We suspect that age was probably an important factor in this difference, as the average age of our patients (84.1 years) was much higher than in the previous study (67.8 years).^15^ Being bedridden appeared to be a very important statistical risk factor for onychomycosis, with an odds ratio of 18.0. We believe that once again, this high risk relates to the cascade of systemic problems associated with bedridden status, of which onychomycosis is simply another by-product. No statistical correlation between diabetes and onychomycosis prevalence was found during our study, which is in agreement with previous knowledge.^14^

Dermatitis may be divided into numerous subcategories such as allergic and stasis dermatitis. Dermatitis is known to affect ageing individuals to an increasing extent as the barrier function of the skin decreases with age. The ability of the skin to eliminate potential allergens from its surface is also reduced and this often leads an accumulation of debris.^10^ A simultaneously compromised immune system usually diminishes both inflammatory responses and the systemic defences. In such situations, skin dermatitis may ensue. Dry skin also provides an effective starting point for many of these dermatoses as the compromised skin surface is more easily irritated than moisture-laden dermis. We found a dermatitis prevalence of 8.9% among our elderly patients, which is much lower than previous dermatology studies documenting rates of 22.8% to 58.8%.^3^^,^^10^ As no significant statistical associations could be derived with respect to dermatitis, we are unable to explain why our result was so low. Nevertheless, as more than one-third of all cases were seen on the patients’ back (37.5%), we suspect that hot weather and bed rest are important contributory factors in this condition as they simultaneously provide moisture and pressure to vulnerable skin areas.

Skin cancer is a common dermatological affliction of elderly individuals; particularly basal cell carcinoma and squamous cell cartinoma.^16^ Basal cell carcinomas are often seen on the face and sun-exposed areas of fair skinned people and are usually the result of cumulative ultra-violet radiation exposure.^17^ Skin cancer was recorded among the medical records of 4.9% of the patients we studied. Of these, 78.6% were basal cell carcinoma, 14.3% squamous cell carcinoma, and 7.1% melanoma. A previous study of elderly individuals revealed a very similar skin cancer prevalence rate of 4.4%.^3^ As the lifetime risk for basal cell carcinoma is estimated to be between 28% and 33% in the United States, we believe that 4-5% is possibly a background level for this disease among elderly citizens.^18^

## CONCLUSION

Overall, our research suggests that skin diseases are reasonably common among nursing home patients in Australia. Intrinsic changes within the dermal structures combined with a lifetime of solar radiation predisposes elderly people to a wide variety of skin diseases, many of which were recorded during this study. It also appears that we have documented skin disease among nursing home patients in Queensland, Australia for the first time. Despite the limitations of the medical records available to us, we believe our results may be useful as a baseline in the development of skin care policy by aged care professionals.

## References

[1] Australian Bureau of Statistics. Population by Age and Sex: Australian States and Territories. ABS Canberra, December 1999.

[2] Commonwealth of Australia. Aged and Community Care: Certified Aged Care Facilities. Commonwealth Department of Health and Aged Care, Canberra, 2000.

[3] Beauregard S, Gilchrest BA. A survey of skin problems and skin care regimens in the elderly. Arch Dermatol 1987;123:1638-43. 3688904

[4] Fenske NA, Lober CW. Skin changes of aging: pathological implications. Geriatrics 1990;45:27-35. 2407619

[5] Ghadially R, Brown BE, Sequeira-Martin SM, Feingold KR, Elias PM. The aged epidermal permeability barrier. Structural, functional, and lipid biochemical abnormalities in humans and a senescent murine model. J Clin Invest 1995;95:2281-90. 7738193 10.1172/JCI117919 PMC295841

[6] Masaki H, Tezuka T. The changes of epidermis with aging. Jpn J Dermatol 1986;96:189-193. (in Japanese) 3735720

[7] Fang KT. The changes of the facial skin with aging: the changes in the amount of both the stratum comeum surface water and Filaggrin. Hifu 1988;30:455-60. (in Japanese)

[8] Aisen E, Shafran A, Gilhar A. Sebum and water content in the skin of aged immobilized patients. Acta Derm Venereol 1997;77:142-3. 9111827 10.2340/0001555577142143

[9] Hirschmann JV, Raugi GJ. Adult scurvy. J Am Acad Dermatol 1999;41:895-906. 10570371 10.1016/s0190-9622(99)70244-6

[10] Thaipisuttikul Y. Pruritic skin diseases in the elderly. J Dermatol 1998;25:153-7. 9575676 10.1111/j.1346-8138.1998.tb02371.x

[11] Shenefelt PD, Fenske NA. Aging and the skin: recognizing and managing common disorders. Geriatrics 1990;45:57-66. 2210395

[12] Kemna ME, Elewski BE. A U.S. epidemiologic survey of superficial fungal diseases. J Am Acad Dermatol 1996;35:539-42. 8859279 10.1016/s0190-9622(96)90675-1

[13] Scher RK. Onychomycosis: a significant medical disorder. J Am Acad Dermatol 1996;35 Suppl:S2-5. 8784302 10.1016/s0190-9622(96)90061-4

[14] Rich P. Special patient populations: onychomycosis in the diabetic patient. J Am Acad Dermatol 1996;35 Suppl:S10-2. 8784304 10.1016/s0190-9622(96)90063-8

[15] Siragusa M, Schepis C, Palazzo R, Fabrizi G, Guameri B, Del Graeco S, . Skin pathology findings in a cohort of 1500 adult and elderly subjects. Int J Dermatol 1999;38:361-6. 10369546 10.1046/j.1365-4362.1999.00720.x

[16] Proper SA, Rose PT, Fenske NA. Non-melanomatous skin cancer in the elderly: diagnosis and management. Geriatrics 1990;45:57-65. 2194908

[17] Pariser DM, Phillips PK. Basal cell carcinoma: when to treat it yourself, and when to refer. Geriatrics 1994;49:39-44. 8125351

[18] Miller DL, Weinstock MA. Nonmelanoma skin cancer in the United States: incidence. J Am Acad Dermatol 1994;30:774-8. 8176018 10.1016/s0190-9622(08)81509-5

